# To the Editors of the North-Western Medical and Surgical Journal

**Published:** 1854-01

**Authors:** Thomas Hall

**Affiliations:** Chairman of Committee on Obstetrics


TOULON, STARK CO., ILL.

January 6, 1854.

*To the Editors of the North-Western Medical and Surgical Journal:*

GENTLEMEN:—I have sent you a portion of some remarks on the medicinal use of Turpentine, based on various articles in "Copland's Dictionary of Practical Medicine," and a paper in the last Vol. of Transactions of our State society by Dr. Samuel Thompson. The manner, adopted to convey my ideas on the various subjects that will be introduced, was deliberatively chosen; and I trust the article will be permitted to appear in the journal without mutilation or material change. My motive in writing the paper will plainly appear in the sequel. I will also in this place request your numerous readers to send me an account of any unusual or interesting cases of Midwifery that have occured or may occur in their practice up to the first of next May. The Constitution of our State Society closely confines the Committee on Obstetrics within narrow limits. I shall not hesitate to break over the bounds if by so doing an interesting and practically-useful report can be produced .Every case sent to me shall be duly acknowledged and my thanks cheerfully given.

I remain truly yours

THOMAS HALL, M.D., M.R.C.S., L.A.C.,
*And Chairman of Committee on Obstetrics.*

